# A Charge of Malingering

**Published:** 1911-04-15

**Authors:** 


### Medico-Legal Points.

### A CHARGE OF MALINGERING.

In a case at Stockton it appears that a rivet heater named Nicholas Kenny, of Stokes' Yard, Church Row, Stockton, asked for a compensation award of 8s. per week, which Messrs. Richardson, Duck & Co., shipbuilders, Thornaby, had paid him from November 22, 1909, to February 7, 1910, to be continued until he was able to resume work. The firm stopped payment in February on the ground that applicant had completely recovered. It appeared that the applicant fell from a plank in the shipyard and injured his back.

Applicant, who was not legally represented, said he suffered from pains in the back and stomach, and was unable to work. He denied, in answer to Mr. Reuben Cohen, who represented the respondents, that he had refused to allow Dr. Hughes to examine him.

Dr. D. A. Cameron said he examined the applicant, in company with Dr. Hughes, and could not find any cause for the pains applicant said he was suffering from. He was quite well again.

Dr. H. M. Hughes said when he examined applicant he behaved in a most extraordinary manner, as if he was in great agony. He could hardly walk across the room, and interfered with the examination as much as possible. He was told to lie on the couch, but before witness started to examine him, and, in fact, whilst he was at the other side of the room, applicant began to shout and bellow. In taking his things off applicant appeared as if he could not put his arms up properly, but when he was dressing himself he forgot himself and put his arms above his head, witness telling him to be careful or he would break the gas globe. After leaving the surgery applicant walked away in sprightly style, with a drum-and-fife-band swing. He had been watching applicant in the box, and if he was suffering from half the things he complained of he would be unable to stand. There was no sign of bodily deterioration. He believed he was quite well now.

His Honour dismissed the application.

